# Prediction of Human-Computer Interaction Intention Based on Eye Movement and Electroencephalograph Characteristics

**DOI:** 10.3389/fpsyg.2022.816127

**Published:** 2022-04-12

**Authors:** Jue Qu, Hao Guo, Wei Wang, Sina Dang

**Affiliations:** ^1^School of Aeronautics, Northwestern Polytechnical University, Xi’an, China; ^2^Air and Missile Defense College, Air Force Engineering University, Xi’an, China

**Keywords:** electroencephalograph, eye movement, human-computer interaction, support vector machine, task type and difficulty, intention prediction

## Abstract

In order to solve the problem of unsmooth and inefficient human-computer interaction process in the information age, a method for human-computer interaction intention prediction based on electroencephalograph (EEG) signals and eye movement signals is proposed. This approach is different from previous methods where researchers predict using data from human-computer interaction and a single physiological signal. This method uses the eye movements and EEG signals that clearly characterized the interaction intention as the prediction basis. In addition, this approach is not only tested with multiple human-computer interaction intentions, but also takes into account the operator in different cognitive states. The experimental results show that this method has some advantages over the methods proposed by other researchers. In Experiment 1, using the eye movement signal fixation point abscissa Position X (PX), fixation point ordinate Position Y (PY), and saccade amplitude (SA) to judge the interaction intention, the accuracy reached 92%, In experiment 2, only relying on the pupil diameter, pupil size (PS) and fixed time, fixed time (FD) of eye movement signals can not achieve higher accuracy of the operator’s cognitive state, so EEG signals are added. The cognitive state was identified separately by combining the screened EEG parameters Rα/β with the eye movement signal pupil diameter and fixation time, with an accuracy of 91.67%. The experimental combination of eye movement and EEG signal features can be used to predict the operator’s interaction intention and cognitive state.

## Introduction

As an important part of intelligent human-computer interaction, human-computer interaction intention prediction of the operator according to fuzzy interaction information, and through data mining and analysis can provide cooperative services for operators, so as to improve operator operation efficiency, reduce operational errors, and improve task completion (Dupret and Piwowarski, 2008; Li et al., 2010). At present, intention prediction has been applied in aviation flight operation, weapons and equipment operation, computerized numerical control (CNC) machine tool operation, manufacturing operation system and many other fields, used to solve the operational efficiency and task completion problems of heavy operator interaction task, high information complexity, and large physiological and psychological load (Ganglei, 2021b).

Human-computer interaction intention prediction mainly includes behavioral intention prediction and cognitive intention prediction of operators (Shen et al., 2011; Ganglei, 2021a). The main method of intention prediction is to conduct interaction intention identification by collecting human-computer interaction data and analyzing the data, thus achieving the purpose of interaction intention prediction (Li et al., 2008; Teevan et al., 2008).

Intention is a mental state, a plan or reaction tendency to future behavior. An action is an action executed by an agent that points to its target state, which is envisaged according to the target state it ultimately wants to achieve, even if it may not be achieved in some cases. Cognition is a term referring to the mental processes involved in gaining knowledge and comprehension. In other words, it is the process of information processing of external things acting on people’s sensory organs. It includes feeling, attention, memory, thinking, and other psychological phenomena. Habitually, cognition corresponds to emotion and will.

In terms of behavioral intention prediction, an operation behavior analysis method of inspection robot based on Bayesian network was proposed by Tang et al. (2021). Although the proposed method realizes the function of rapid reasoning about the operation behavior intention of inspection robot, the realized human-computer interaction between robots does not study the human-computer interaction between operators and computers, and the established inspection system for inspection robot operation was subjective. A method of behavior intention recognition for target grabbing was proposed by Zhao et al. (2019). By taking the trajectory of human upper limbs as the main criterion, a user behavior intention model was established. The research on the intention of human-computer interaction between people and computers is realized. This behavior intention integrates the user’s upper limb trajectory into the prediction model, but there is some subjectivity in the modeling process. A multi-information fusion network architecture of human motion intention recognition combining eye movement information, position and attitude information, and scene video information was proposed by Zhang et al. (2021), which effectively reduced the subjectivity of prediction, but the research on user’s behavior intention was relatively simple. Action prediction for a full-arm prosthesis using eye gaze data was proposed by Krausz et al. (2020). A system for controlling the up-and-down movement of artificial limb by using single-channel electroencephalograph (EEG) signal was developed by Haggag et al. (2015), which is convenient and accurate to operate. Yao et al. (2018) combined EEG and eye movement signals to identify different action imagination modes of the same limb, focusing on verifying that the recognition accuracy of EEG combined with eye movement signals is higher than that of a single EEG signal.

The EEG signals are often used in psychological and medical research (Caixia et al., 2021), and also in cognitive intention prediction. The operator’s cognitive intention was successfully predicted by Lu and Jia (2015) and others by visualizing the eye movement data collected during the experiment and using four learning algorithms. A set of eye movement index system to evaluate cognitive load was established by Chen et al. (2011), which realized the prediction of operators’ cognitive intention. Ahern and Beatty (1979) found that with the increase of task difficulty and cognitive load, the pupil diameter will increase. Singh et al. (2020) realized the recognition of online operators’ cognitive intention by using the eye fixation characteristics. By combining EEG signals and eye movement signals to identify the emotions of operators in human-computer interaction system, the components of EEG signals and eye movement signals that have great influence on emotion identification were identified by Lu (2017). Park et al. (2014) explored the influence of EEG and eye movement signals on operators’ implicit interaction intention in the process of interaction, and the experimental results showed that the combination of EEG and eye movement signals was more accurate than a single physiological signal.

In conclusion, intention prediction using behavioral data and physiological indicators is the main research direction of human-computer interaction intention prediction. Behavioral data are predicted through the operator’s past operation experience and do not consider the randomness, universality, and real-time data processing during the operation, so the established intention prediction method has certain subjectivity. Therefore, physiological metrics are commonly applied to behavioral prediction and cognitive prediction. The existing indicators mainly focus on the study of eye movement indicators and EEG indicators. The intention prediction model established based on this one can objectively identify the operation intention of the operator and establish a more accurate intention prediction model.

In recent years, many scholars have also combined eye movement and EEG signals to explore the human-computer interaction intention of operators. Although Wei et al. (2021) also studied the prediction of human-computer interaction intention by combining EEG and eye movement characteristic signals, they focused on demonstrating that the prediction of human-computer interaction intention by using eye movement and EEG characteristic data is more accurate than that by using eye movement data alone, and did not study the prediction performance of specific eye movement and EEG characteristic indicators and their combinations.

This article integrates eye movement and EEG indexes, identifies real-time interaction intention for specific task types and task difficulty, selects significant difference indicators by one-way ANOVA, selects indexes by support vector machine (SVM), constructs task type and difficulty, and compares the prediction results of the prediction method.

## Methodology

### Subjects and Equipment

#### Subjects

A total of 50 college students (25 males and 25 females), aged 19 years old, ∼25 years old, right-handed, and no cognitive impairment were recruited. No central nervous system (CNS) diseases were found in the examination and the EEG showed no abnormalities. Both naked visual acuity and corrected visual acuity were above 5.0. The subjects should rest fully before the start of the experiment to avoid strong reactions and maintain emotional stability. At the same time, the hair should be washed clean and cut short. Medicine should not be taken within 24 h before the start of the experiment. Drinking tea and coffee was not permitted so that it does not to affect the reliability of relevant physiological parameters during the experiment.

#### Experimental Equipment

**FIGURE 1:**
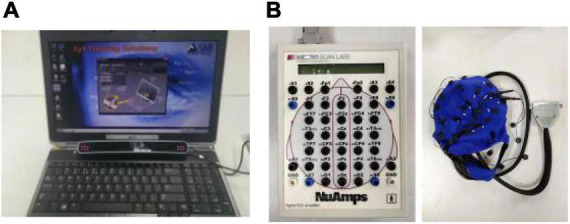
Physiological signals acquisition equipment. **(A)** Eye movement equipment. **(B)** Electroencephalograph (EEG).

The experimental equipment mainly includes SMI-RED eye motor as shown in Figure 1A. The maximum sampling frequency is 250 Hz. Neuroscan-NuAmps EEG, as shown in Figure 1B, with a maximum sampling frequency of 1,000 Hz, is used to collect EEG signals during the experiment.

### Experimental Design About Task Type and Difficulty

In order to reasonably summarize the operator’s operation state in the process of human-computer interaction, this article defines the operator’s operation type and state as five operation types and three operation states in the experiment, combining with the human-computer interaction process in important and complicated fields such as air traffic control.

#### Task Type Setting

**FIGURE 2:**
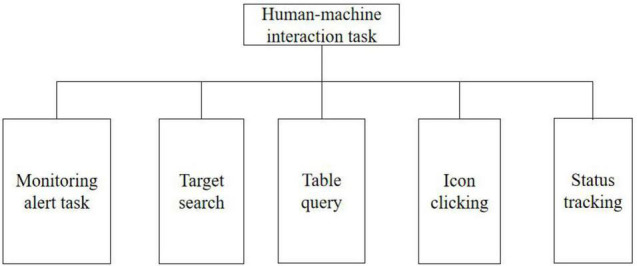
Five kinds of human-computer interaction tasks.

**FIGURE 3:**
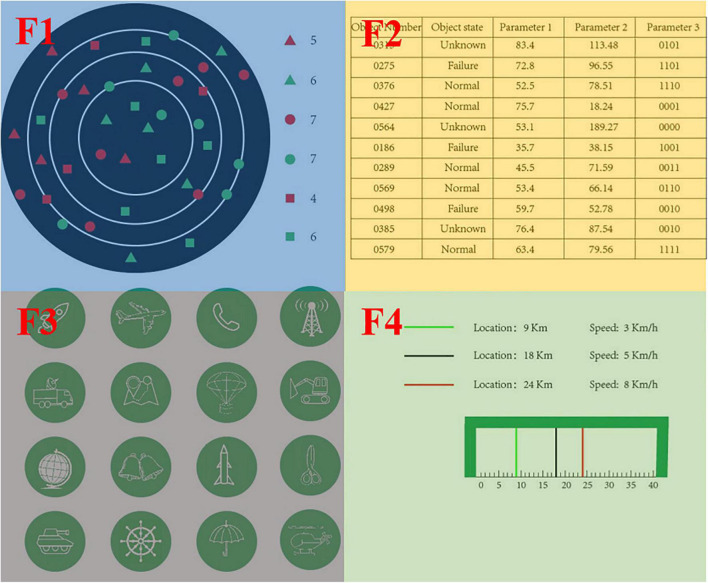
Experimental interface.

During the experiment, the subject needs to complete the designated operational task in accordance with the requirements of the experiment. In Experiment 1, the subjects need to complete five different experimental tasks. In order to reasonably sum up the operator’s intention of human-computer interaction in the process of operating the computer, this article defines the operator’s operation tasks in the process of human-computer interaction as five types as shown in Figure 2, combining with the human-computer interaction process in important and complicated fields such as air traffic control. The operation interface is shown in Figure 3; the top left ribbon (F1) is the target search task interface, and the induced interaction intention is a target search; the top right ribbon (F2) is the table query task interface, and the induced interaction intention is a table query; the lower left ribbon (F3) is the icon clicking the task interface, and the induced interaction intention is the icon clicks; the lower right row (F4) is the status tracking task interface, and the induced interaction intention is the target tracking. The whole scene (F0) is the monitoring alert task interface, and the induced interaction intention is monitoring alert.

“Monitoring alert” means that the operator does not need to complete any human-computer interaction tasks, but only needs to pay attention to the changes of main parameters on the interface and monitor whether there is any abnormality.

“Target search” refers to the process that the operator needs to find out a specific target in the background containing interference icons.

“Table query” refers to the process that the operator needs to query the required information in the table containing the target status parameters.

“Icon click” refers to the process that operators need to click an icon button to trigger corresponding instructions and complete specific operations.

“State tracking” refers to the process that operators need to pay real-time attention to the specific parameters of specific targets and judge whether the operation time is ripe or not.

#### Difficulty Gradient Design Based on Task Type

Each of the five tasks can be divided into three task difficulties.

**FIGURE 4:**
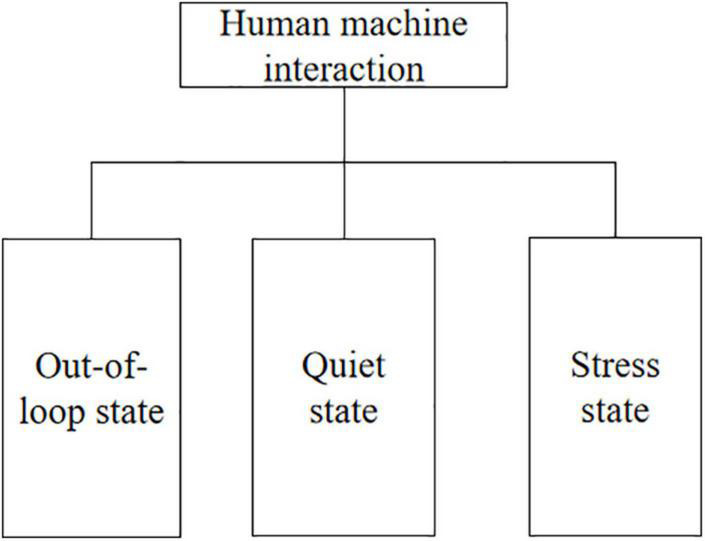
Three operating states of human-computer interaction.

In the second experiment, the subjects need to perform tasks in three special cognitive states, as shown in Figure 4.

“Out-of-loop state” refers to the phenomenon that the operator may lose attention during long-term monotonous and boring interactive tasks, which can also be understood as what we usually call “distracted.”

“Quiet state” refers to the state in which the operator normally performs daily interactive tasks, which is similar to the “monitoring alert” mentioned in Experiment 1.

“Stress state” refers to the state in which the operator is at a loss when he/she is under great pressure and when he/she is in an abnormal situation during the execution of a stressful task.

**FIGURE 5:**
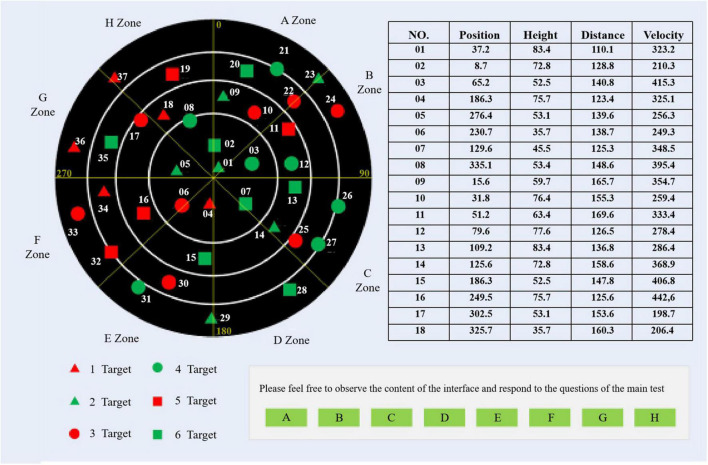
Normal state experimental interface.

**FIGURE 6:**
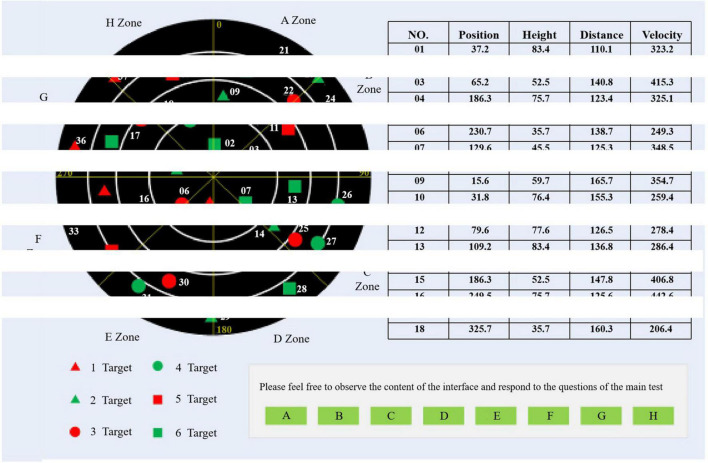
Stress state experimental interface.

The task interface of the second experiment induced a cognitive state as shown in Figure 5, which can be divided into four regions as the “radar monitoring task” as the background. The top left is the target search area, in which six kinds of targets move randomly, and concentric circles and angle lines can roughly show the distance and orientation information of targets. On the upper right is the state parameter area. All targets in the target search area can find accurate state parameters in the table. You can turn pages with the mouse. At the bottom left is the legend area, which shows the category information of icons in the target search area. On the lower right is the operation area, where the subjects perform corresponding operations according to the prompts in this area, and click the relevant buttons. The experimental interface under stress is shown in Figure 6.

#### Experimental Process

**FIGURE 7:**
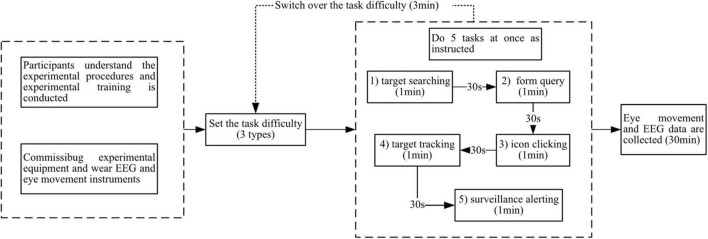
Experimental process.

In the first experiment, in order to collect physiological data with five interaction intentions, all the subjects need to complete five experimental tasks according to the experimental flow shown in Figure 7.

Before starting the experiment, first introduce the experimental tasks and specific requirements to the subjects, and at the same time, there should be some practice operations to ensure that the subjects have a systematic understanding of the experimental tasks, and then calibrate the eye tracker and wear the electrode cap. All the subjects need to complete five experimental tasks as follows:

Monitoring and alert interaction task: Under this task, all elements of the whole interface will change randomly, and the subjects can pay attention to the content they are interested in at will, and report the specific situation of an element in the interface in time, so as to make the subjects in the monitoring and alert interaction state. The task will be completed in about 1 min, and then the next task will be carried out after a short rest.

Target search interactive task: Under this task, the subjects need to search for specific characters in the function F1 whose character positions change randomly, and judge and report whether the number of characters listed on the right is correct, so that the subjects can be in the target search interactive state until the task of judging all the characters is completed, and then take a short rest and proceed to the next task.

Table query interaction task: Under this task, the content presented by the table in function area F2 is constantly updated. After the main test issues the query task, the subjects will try their best to find the corresponding data and report in time, in order to make the subjects in the form query interaction state, have a little rest after completing ten queries, and then carry out the next task.

Click interactive task: Under this task, the target icon that the subject needs to click will be displayed on the screen. Subjects need to complete the search and click on a large number of icons in functional area F3. The system will judge whether the subject clicks correctly, rest after clicking ten times, and then start the next task.

Interactive task of state tracking: Under this task, the position change of the target (black line segment) on the coordinate axis will be displayed in functional area F4, and the subjects are required to pay attention to the state of the target in real time, and answer the questions of the examiners about the state of the target at any time, so as to keep the subjects in the state tracking state, and the duration of this task is about 1 min.

During the experiment, the eye monitor and the electroencephalonete will automatically collect and save the physiological signals to provide the data for the subsequent analysis and processing. It is worth noting that, because one experiment lasts for a long time, the manual labeling time error generated by performing the time synchronization during signal acquisition is negligible.

Experiment 2 subjects were needed to perform the corresponding task manipulation in three different cognitive states.

The experimental task of calm state requires the participants to complete the normal operation. For example, select the target number they are interested in in the target search area, then query the specific parameters in the state parameter area, and respond to the questions of the main test timely. The task lasts about 1 min, and the next task is carried out after a little rest.

The experimental task in the off-loop state does not require any interactive operation of the subjects, but recalls the learning task in the past period, which also lasts for 1 min, and then goes on to the next task after a short rest.

Under the experimental task of stress state, the interface information is difficult to display normally, and the information is more difficult to obtain. Specifically, the target search area and the state parameter area in the experimental interface will produce the random flashing stripes as shown in Figure 6. However, at this time, the subjects still have to perform an intensive interaction task, such as finding the area of multiple specific numbered targets in the target search area, and clicking the relevant button in the operation area, respectively.

## Experiment 1

### Experimental Purpose

A visual interaction experiment that can induce 5 kinds of interaction intention, use eye movements and EEG instruments to collect eye movement characteristics and EEG signals under different interaction intentions, realize the discrimination of different interaction intentions through the classification algorithm, compare the classification effect of eye movement characteristics and EEG signals, and screen out better distinguishing characteristic indicators was designed.

### Results

Due to the large differences in the processing methods of eye movement data and EEG signals, they should be discussed and analyzed separately. First, the eye movement indicators and EEG parameters with good classification effect were selected through differential analysis, and then the characteristic combination with the best discriminative effect was further explored.

#### Analysis of Eye Movement Data Processing

**FIGURE 8:**
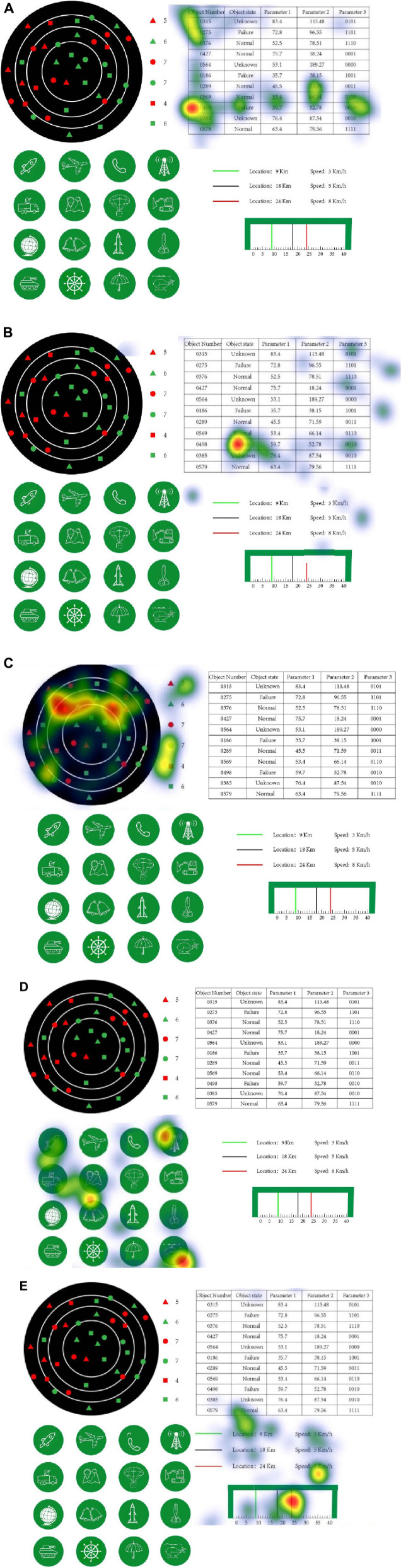
Hot map of subjects in normal state. **(A)** Table query interaction task. **(B)** Monitoring and alert interaction task. **(C)** Target search interactive task. **(D)** Click interactive task. **(E)** Interactive task of state tracking.

In the normal state, the hot maps of subjects in one set of experiments are shown in Figure 8.

As can be seen from Figure 8, the subject eye stay area greatly concerns the task completed. When completing a task, the subjects point will focus in the corresponding task area and focus on the data or status of the task target. In the table search task, the subjects focus on the F2 area and watch the most on the target number 0498; in the monitoring alert task, the fixation points are distributed in many areas, but ultimately in the F2 area; the target is in the F1 area, and the hot spots are relatively evenly distributed, probably because the target itself is scattered, the target will focus on the red line.

(1) Selection of characteristic components of eye motion.

The SMI RED5 eye moving instrument used in this article can measure dozens of eye movement parameters. In order to improve the efficiency of classification and identification, it is necessary to select better differentiated eye movement indicators through differential analysis. Based on the preliminary analysis results of the BeGaze software, the average pupil diameter (Average Pupil Size [APS]), fixation point abscissa mean (Average Position X [APX]), fixation point longitudinal mean (Average Position Y [APY]), average saccade amplitude (ASA), average saccade speed (Average Saccade Velocity [ASV]), and average fixation time AFD (Average Fixation Duration [AFD]) were selected for this experiment. The results of data processing by one-way ANOVA are shown in Table 1.

**TABLE 1:** Analysis results of eye movement index differences.

	APS (mm)	APX (px)	APY (px)	ASA (°)	AAV (°/s)	AFD (ms)
Surveillance alert	4.06	831	363	3.37	29.4	269
Target search	4.25	426	239	4.28	13.7	318
Table query	4.12	1209	247	3.96	19.6	376
Click icon	3.98	449	658	2.39	17.5	326
Status tracking	4.03	1189	671	1.75	5.37	718
*F*	3.83	34.59	32.78	21.84	4.95	17.55
*p*	0.159	0.027*	0.018*	0.000**	0.072	0.068

“*” in the table represents significant differences, and “**” is extremely significant.

Table 1 shows significant differences in APX [*F*_(4,115)_ = 34.59, *p* < 0.05], APY [*F*_(4,115)_ = 32.78, *p* < 0.05], and ASA [*F*_(4,115)_ = 21.84, *P* < 0.05], while APS [*F*_(4,115)_ = 3.83, *p* > 0.05], AAV [*F*_(4,115)_ = 4.95, *p* > 0.05], and AFD [*F*_(4,115)_ = 17.55, *p* > 0.05] differ significantly. Therefore, this article considers that the fixation point X coordinate PX, fixation point Y coordinate PY, and SA can be used to distinguish the interaction intention at this moment.

After the eye motion index screening is completed, it is necessary to choose the eye motion characteristic components for the interaction intention discrimination. The key is to select the number of sampled gaze points. To avoid the stochasticity of individual sampled fixation points, drawing on the treatment of literature (Fan et al., 2016), in this article, PX, PY, and SA of three consecutive sampled fixation points, namely the nine components shown in Table 2, were used as eye movement feature parameters for determining the interaction intention.

**TABLE 2:** Eye movement characteristic component.

	Abscissa *PX*	Ordinate *PY*	Saccade amplitude *SA*
Sampling fixation points *i-1*	*PX*_*i–1*_	*PY*_*i–1*_	*SA*_*i–1*_
Sampling fixation points *i*	*PX*_*i*_	*PY*_*i*_	*SA*_*i*_
Sampling fixation points *i+1*	*PX*_*i+1*_	*PY*_*i+1*_	*SA*_*i+1*_

*Specifically, both PX and PY represent the cross-ordinate values of this sampled fixation point, while SA represents the saccade distance between the sampled gaze point and the previous sampled fixation point.*

(2) Identification results of eye movement characteristics.

**FIGURE 9:**
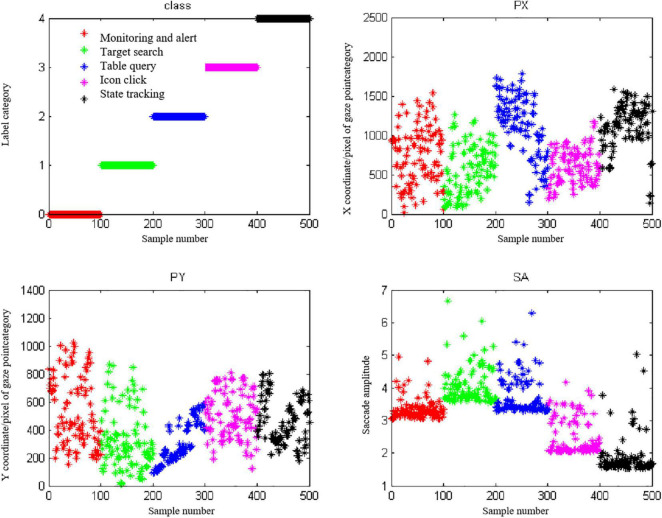
A total of 500 Distribution of typical data.

The training set of this experiment consists of 300 typical data (60 selected each under 5 interaction intentions). The test set consists of 200 typical data (40 selected each under 5 interaction intentions). It uses SVM algorithm and sets category label as (0,1,2,3,4). The SVM algorithm was used to train eye movement features and the parameters of the SVM algorithm were determined by the cross-contrast SVM method. Figure 9 shows the distribution diagram of 500 typical data of PX, PY, and SA.

As can be seen from Figure 9, under the five kinds of interaction intentions, there is a large number of cross-overlap in the range of the three kinds of indicators, which makes it difficult to effectively distinguish the five interaction intentions for a single indicator. Therefore, it needs to be more accurate through the combination of eye movement indicators. Considering that PX and PY represent the positional information of the sampled fixation points, this experiment was analyzed as a whole.

The MATLAB classification results for feature combinations of “PX and PY,” and “PX, PY, and SA” are as follows:

PX and PY:

Accuracy = 76.00%(152/200) (classification);

PX, PY, and SA:

Accuracy = 92.00% (184/200) (classification).

**FIGURE 10:**
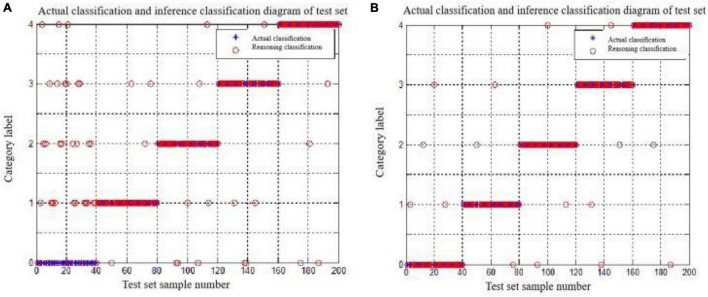
The discriminant classification results for the test set. **(A)** “PX and PY.” **(B)** “PX,PY and SA.”

Figures 10A,B show the specific results of the combination discrimination classification of “PX and PY,” and “PX, PY, and SA,” respectively. The discriminant classification was shown in Table 3. Obviously, the former has a very poor classification effect on monitoring alert, seriously misjudged the monitoring alert as the other four intentions, and has a good classification effect on the other four intentions. After the addition of the saccade range SA, the discrimination accuracy of the monitoring alert is significantly improved, and the classification effect of the other four intentions has also been strengthened to a certain extent, which shows that the saccade range directly affects the inference results of the surveillance alert. At the same time, there are a few cases in which target search, table query, icon click, and status tracking are misjudged as surveillance alert. The reason may be that the location parameters PX and PY of the gaze point in the surveillance alert state overlap with the other four states.

**TABLE 3:** The discriminant classification results for PX and PY.

PX and PY		Forecast				
		Mission 1	Mission 2	Mission 3	Mission 4	Mission 5
Actual	Mission 1	13	1	3	3	2
	Mission 2	10	36	2	2	0
	Mission 3	8	1	33	0	1
	Mission 4	6	2	1	34	1
	Mission 5	3	0	1	1	36

#### Analysis of the Electroencephalograph Signal Processing

(1) Preprocessing of EEG signals.

The EEG signals have strong individual variability, so the commonly used characteristic indicators are the proportion of average power to total power in each frequency segment, or the ratio of average power between different frequency bands, such as (α + β)/θ,α/β, (α + θ)/β, etc. But these indicators also have certain individual differences (Alazrai et al., 2019; Pei et al., 2018). In this paper, the collected EEG signals are first denoised by wavelet transformation, and then obtained by power spectral analysis. The average power of different frequency bands is calculated, and the corresponding ratio can be obtained after simple calculation. It is worth noting that, due to the complex processing of EEG signals, the average power of the basic time period is calculated at 0.5 s (In reference to Gu et al., 2016; Liu et al., 2021). From the experimental process, the duration of the five interaction tasks was above 1 min, so each interaction state has at least 120 sets of data that can meet the basic requirements of the classification algorithm.

**FIGURE 11:**
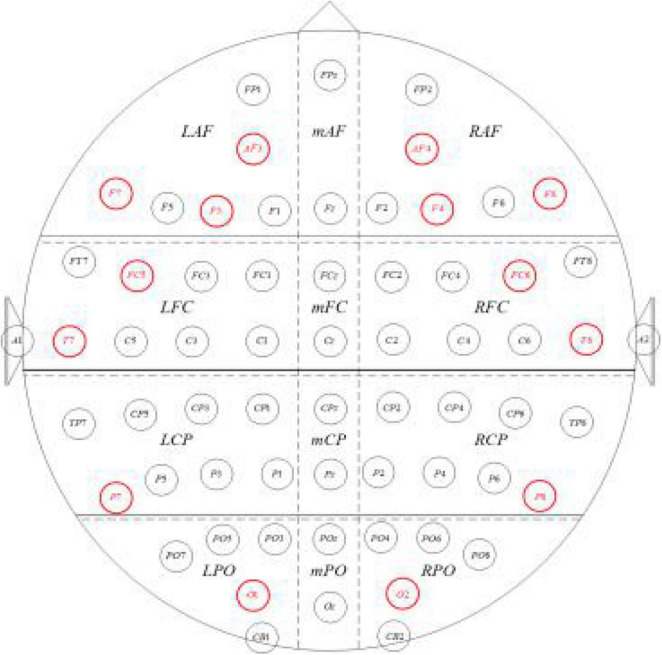
Schematic location of the 14 conductive electrodes selected.

The electrodes on the EEG instrument used in this experiment have been installed according to the 10–20 standardized electrode guidance method, but in order to improve the efficiency of the EEG signal processing and make the research results more generalized, the representative guides should be selected for processing and analysis. This experiment adopted the recommendations of the American EEG Number Association standards, and 14 guides including F7, F8, P7, and P8 as shown in Figure 11 were selected as the EEG signal acquisition channels.

**FIGURE 12:**
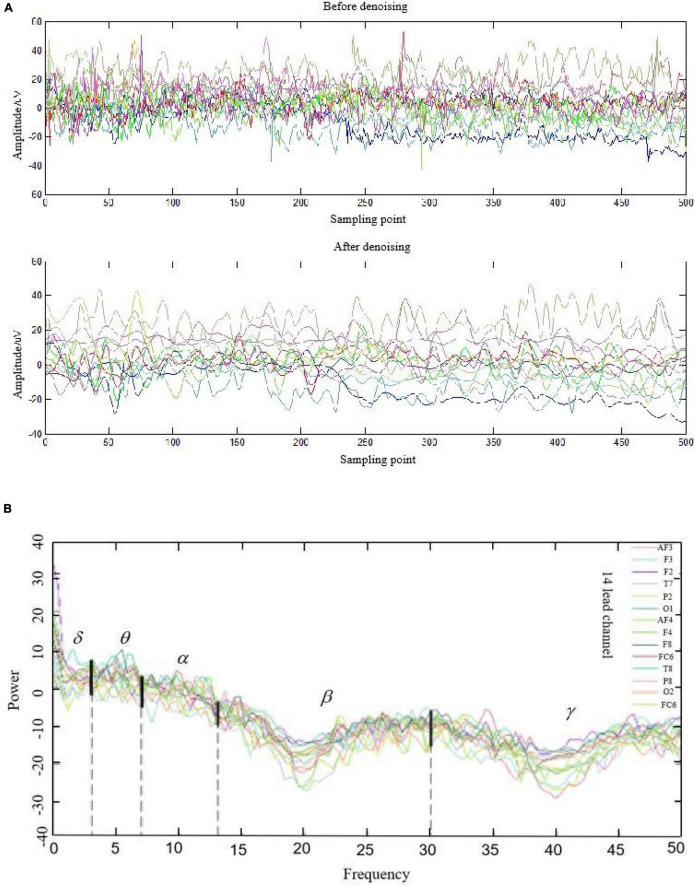
Preprocessing results of 14-lead EEG signals of a subject. **(A)** Denoising treatment. **(B)** Power spectrum analysis.

Denoising processing, power spectrum analysis of EEG signals in the middle of the target search interaction task, and the results are shown in Figure 12. It can be found that the effect of denoising is relatively ideal, and the power density curve of different channels has different degrees, but it shows a similar trend: the power density of slow wave (δ,θ) is relatively stable on the whole, while the power density of fast wave (β,γ) has large fluctuations. Meanwhile, intuitively seen from the power spectral density, the power density of the slow wave in this time period is significantly higher than that of the fast wave.

(2) Differential analysis of the EEG indexes.

In this experiment, 8 commonly used index parameters such as R_δ_, R_θ_, R_α_, R_β_, and R_γ_ and different frequency bands such as R_α/β_, R_θ/β_, and R_(α +θ)/(α +β)_ were selected, and one-way ANOVA was performed, and the results are shown in Table 4. The average power is obtained as shown in formula (1):


(1) $$A_{\alpha }=\frac{\int_{a}^{b}P\,\left(f\right)\,df}{b-a}$$

**TABLE 4:** Results of EE parameters (first 5 rows:%).

	R_δ_	R_θ_	R_α_	R_β_	R_γ_	R_α/β_	R_θ/β_	R_(α+θ)/(α+β)_
Surveil-lance alert	27.50	23.77	17.56	20.96	10.20	83.74	113.39	107.29
Target search	19.24	15.61	25.38	32.40	7.37	78.33	48.16	70.93
Table query	18.75	21.40	28.49	26.07	5.29	109.31	82.12	91.46
Click icon	11.07	26.69	19.78	13.93	28.53	142.02	191.68	137.88
Status tracking	15.53	19.58	27.58	23.49	13.81	117.41	83.36	92.35
F	18.95	21.18	23.73	23.69	13.04	60.16	37.41	49.53
p	0.201	0.094	0.197	0.253	0.062	0.051	0.214	0.149

Where A_α_ is the average power of the frequency band α, (a, b) is the upper and lower limits of the frequency band α, P (f) is the power spectral density of the frequency band α, and the average power method of the remaining frequency bands is similar to this. In addition, the meaning of the R_α/β_ value is the average power ratio between the frequency band α and the frequency band β, and the meaning of the R_α_ value is the average power ratio of the frequency band α to the total frequency band, which is equivalent to the R_(α/(δ +θ +α +β +γ)_. The specific meaning of the other statements will not be repeated here.

From Table 4, the eight kinds of EEG parameters selected in this experiment in the difference of interaction intention is not obvious (*p* > 0.05), so it is difficult to realize the effective discrimination of interaction intention. The reason may be that the power ratio of different frequency bands is difficult to interpret the interaction intention. Whether other EEG features can successfully distinguish the interaction intention defined in this paper needs further exploration. As for other EEG characteristics can successfully distinguish the interaction intention defined in this article remains to be further explored. Therefore, the discrimination of interaction intention in this article is mainly based on the eye movement features, and the feature combination of “PX, PY, and SA” will be used for the real-time discrimination of interaction intent below. The discrimination dassifivation was been shown in Table 5.

**TABLE 5:** The discriminant classification results for PX, PY, and SA.

P X, PY and SA		Forecast				
		Mission 1	Mission 2	Mission 3	Mission 4	Mission 5
Actual	Mission 1	36	1	1	1	1
	Mission 2	2	37	1	2	0
	Mission 3	1	1	37	1	1
	Mission 4	1	1	0	36	0
	Mission 5	0	0	1	1	38

## Experiment 2

### Experimental Purpose

Finally, through the experiment, the good effect of the eye movement parameter characteristics predicted by the operator is determined. However, in practice, the interface of electronic products will become more and more complex, and sometimes unexpected situations will occur in the interactive environment. Under this condition, the operator’s pressure will increase, which will directly affect the operation efficiency. In view of this problem, we envisage to monitor the state of the time of the operator’s operation in real time, giving timely reminders and providing effective decision assistance at the beginning of the “negative state” to ensure the reliability of the human-computer interaction process. Therefore, this section has designed interaction experiments to induce different cognitive states and to distinguish the operational state of the operator through a classification algorithm.

### Results

#### Analysis of Eye Movement Data Processing

(1) Selection of characteristic components of eye motion.

After the initial screening of the BeGaze software, in this experiment, the average pupil diameter APS, ASA of mean saccade amplitude, mean saccade velocity ASV, average gaze time AFD, and mean blink time (Average Blink Duration [ABD]) were selected for one-way ANOVA. The results are shown in Table 6.

**TABLE 6:** Results of the differential analysis of eye movement indicators.

	APS(mm)	ASA(°)	AAV(°/s)	AFD(ms)	ABD(ms)
Quiet state	3.95	3.69	27.8	237	186
Out-of-loop state	4.47	4.17	17.8	276	213
Stress state	5.06	4.49	26.9	201	141
F	6.37	11.36	13.73	4.78	17.63
p	0.000**	0.108	0.083	0.039*	0.143

“*” in the table represents significant differences, and “**” is extremely significant.

Table 6 shows significant differences between APS [*F*_(2,69)_ = 6.37, *p* < 0.05] and AFD [*F*_(2,69)_ = 4.78, *p* < 0.05], but not between ASA [*F*_(2,69)_ = 11.36, *p* > 0.05], AAV [*F*_(2,69)_ = 13.73, *P* > 0.05], and ABD [*F*_(2,69)_ = 17.63, *p* > 0.05]. Therefore, this article suggests that the pupil diameter PS and fixation time FD at a certain time can be used to distinguish the cognitive state at this moment. Similarly, we selected the PS and FD from three continuously sampled fixation points, with a total of six components, as the eye movement feature parameters distinguishing the cognitive state.

(2) Identification results of eye movement characteristics.

**FIGURE 13:**
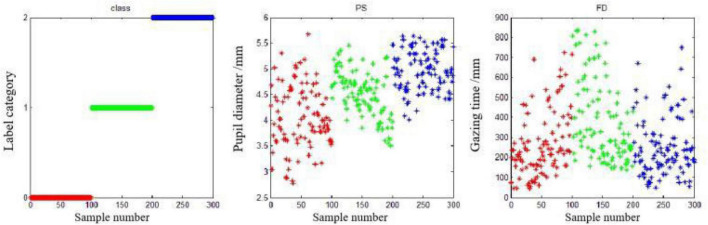
A total of 300 typical data distribution plots of the groups.

The training set of this experiment consists of 180 typical data (60 selected for each of 3 cognitive states). The test set consists of 120 typical data (40 selected for each of 3 cognitive states) for data processing using SVM and sets the category label to (0,1,2). Figure 13 shows the distribution map of 300 typical data sets, PS and FD. Obviously, there is a considerable cross-overlap between the interval ranges of the two indicators, which makes it difficult to effectively distinguish the three cognitive states for a single indicator. Therefore, a more accurate distinction needs to be achieved through the combination of the two.

The MATLAB runs combined with PS and FD were:

PS and FD: Accuracy = 74.17% (89/120) (classification).

**FIGURE 14:**
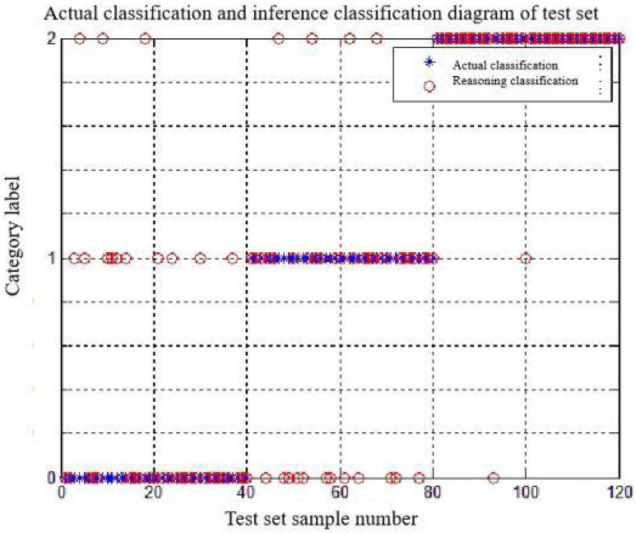
The discrimination classification results for the test set.

From the results, the accuracy of the discriminant classification is relatively low, and Figure 14 shows the specific classification results of the “PS and FD” features in combination. The discrimination dassifivation was been shown in Table 7. Obviously, the stress state of misjudgment is less, identification effect is ideal, but the identification of calm state and ring state effect is poor, ring state for calm state and calm state for ring state, so this experiment to further explore the EEG parameter can achieve the accurate discrimination classification of the two.

**TABLE 7:** The discrimination classification results for the test set.

PS and FD		Forecast		
		Difficulty 1	Difficulty 2	Difficulty 3
Actual	Difficulty 1	31	4	1
	Difficulty 2	6	23	1
	Difficulty 3	3	13	38

#### Analysis of the Electroencephalograph Signal Processing

The EEG signals of this experiment were processed according to the preprocessing method in Experiment 1, and the one-way ANOVA results of the 8 commonly used EEG index parameters are shown in Table 8.

**TABLE 8:** Results of EEG parameters (units in top 3 rows:%).

	R_δ_	R_θ_	R_α_	R_β_	R_γ_	R_α/β_	R_θ/β_	R_(α +θ)/(α +β)_
Quiet state	29.70	14.11	13.83	16.06	26.31	86.10	87.85	93.47
Out-of-loop state	24.22	15.18	16.33	22.67	21.61	72.04	66.99	80.81
Stress state	23.80	15.13	13.72	22.86	24.48	60.03	66.21	78.89
F	8.76	15.31	17.85	20.36	19.87	19.35	28.51	37.48
p	0.108	0.089	0.383	0.225	0.076	0.000**	0.214	0.148

“**” is extremely significant.

From Table 8, the difference of R_α/β_ [*F*_(2,69)_ = 19.35, *p* < 0.05] is significant, and none of the remaining seven EEG parameters is obvious (*p* > 0.05). Therefore, this article believes that the EEG parameter R/for a certain period can be used to distinguish the cognitive status of this period. Similarly, whether other EEG features can successfully distinguish the cognitive states divided in this article needs to be further explored. At the same time, in the three cognitive states, a single EEG index R_α/β_ also has a relatively serious cross-overlapping phenomenon, and it is difficult to accurately complete the discrimination of the cognitive state. Therefore, we will examine the effect of combining ocular movement features and EEG parameters in classifying cognitive states, and explore whether the combinatorial features can achieve accurate discrimination of the “out-of-loop state” and the “calm state.”

#### Classification Results of the Combined Features

Because the EEG parameters were sampled at 0.5 s as a basic time period, the number of samples for the EEG measures R_α/β_ was at least 120 for each cognitive state. Based on the experiment-like library, we selected 100 typical EEG parameters from the intermediate periods to join the test and training sets, respectively. There are three combinations of eye movement features and EEG parameters: “R_α/β_ and PS,” “R_α/β_ and FD,” and “R_α/β_, PS, and FD,” and the MATLAB operation results are, respectively:

Rα/βand PS: Accuracy = 80.83% (97/120) (classification);

Rα/βand FD: Accuracy = 77.50% (93/120) (classification);

Rα/β, PS, and FD: Accuracy = 91.67% (110/120) (classification).

**FIGURE 15:**
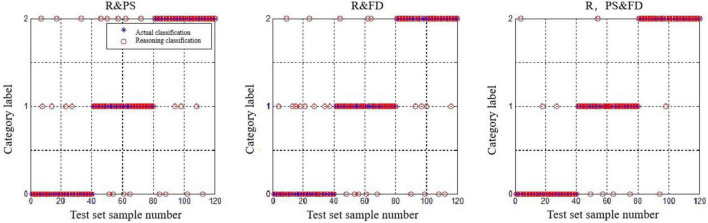
Discriminant classification results of the test set.

Obviously, the combination features of eye motion features and EEG parameters have higher accuracy than the eye motion features, and Figure 15 shows the specific classification results of the three feature combinations of “R_α/β_ and PS,” “R_α/β_ and FD,” and “R_α/β_, PS, and FD,” respectively. The discrimination dassifivation was been shown in Table 9.

**TABLE 9:** Comparison of results of three combinations.

R and PS		Forecast		
		Mission 1	Mission 2	Mission 3
Actual	Mission 1	33	4	4
	Mission 2	4	21	3
	Mission 3	3	5	33

**R and FD**		**Forecast**		
		**Mission 1**	**Mission 2**	**Mission 3**

Actual	Mission 1	2	3	4
	Mission 2	8	32	4
	Mission 3	30	5	32

**R, PS, and FD**		**Forecast**		
		**Mission 1**	**Mission 2**	**Mission 3**

Actual	Mission 1	37	4	1
	Mission 2	2	35	1
	Mission 3	1	1	38

Figure 15 shows that the combination of EEG parameters and single movement features significantly reduces the misjudgment of desyclic and calm states compared with the combination of PS and FD, but partly affects the recognition of stress states. Under the feature combination of “R_α/β_, PS, and FD,” the misjudgment of the three cognitive states is significantly reduced, and the identification effect is relatively ideal. Therefore, this feature combination can be used for real-time discrimination of the operation status of operators in the process of human-computer interaction.

There are many classification methods for EEG data and eye movement data. The algorithms widely used in EEG signals and classification include K-nearest neighbor, quadratic discriminant analysis, decision tree, and SVM method, and eye movement data classification include SVM method, K-nearest neighbor, and Fisher linear discrimination. To validate the effect of the method used in this article, the extracted EEG feature vectors were fed into the above classifier, and the classification performance is shown in the table. Comparing the accuracy, precision, recall, and F1 values of each classifier, the SVM classification method used can be better than the above classification K-nearest neighbor, secondary discriminant analysis and decision tree are widely used in the classification of EEG signals. In order to verify the effect of this method, the extracted EEG feature vector is input into the above classifier, and the classification performance is shown in Table 10. Comparison of accuracy, precision, recall, and F1 values of each classifier shows that the SVM classification method used in this article outperforms the K-nearest neighbor algorithm.

**TABLE 10:** EEG classification performance (%) for different classifiers.

Item	Accuracy	precision	Recall	F1 value
K-nearest neighbor	87.50	86.86	90.10	87.68
SVM	93.27	92.63	95.77	96.10

## Summary of Experimental Results

The results obtained from comprehensive experiments 1 and 2 show that the proposed method to identify human-computer interaction intent based on EEG signals and eye movement signals is effective. According to the five interaction intention discrimination of operators, the EEG signals overlap greatly during classification. At this time, the abscissa of eye movement signal gaze point PX, gaze point ordinate PY, and saccade amplitude SA have great advantages, and the accuracy of interaction intention recognition has reached 92%.

For the cognitive status discrimination of operators in special cases, Experiment 2 added EEG signals with a single use of eye movement signal pupil diameter PS and fixation time FD (S) to achieve a high accuracy rate of operator cognitive status (Fixation Duration). The cognitive states were identified separately by combining the screened EEG parameters R_α/β_ with the pupil diameter of eye movement signals and gaze time, and the R_α/β_, PS, and FD combinations achieved an optimal accuracy of 91.67%. The EEG emotional features and image visual features were fused by Liu et al. (2021) for emotion recognition. This method has been verified on face of China emoticon picture system, and it is found that the average recognition accuracy of seven emotions is 88.51%. A multimodal fusion method based on attention and joint attention was proposed by Kim (2020), which uses eye movement signals to identify emotions, and the highest accuracy rate is 82.7%. A data fusion method based on EEG and eye movements was proposed by Wei (2019), which improved the accuracy of recognition and prediction of motion imagination. The average classification accuracy of feature layer and decision layer reached 81.16 and 82.56%, respectively. The identification method of human-computer interaction intention proposed in this article is obviously more effective and comprehensive.

## Conclusion

In order to solve the contradiction between the limited attention, energy, reaction power and psychological bearing capacity of operators during human-computer interaction and the huge information quantity, fast update, and complex variety, this article proposes a method to identify human-computer interaction intention and cognitive state based on eye movement and EEG signals. The experimental results show that the method proposed in this study predicts human-computer interaction intention driven by higher accuracy.

Experimental one by design specific human-machine interaction operation task, using eye movement and EEG equipment to collect operator eye signals and EEG signals, using one-way ANOVA to screen the collected data, and then use SVM algorithm to test and train the screening data, and finally get the data classification and the accuracy of interaction intention discrimination. As in Experiment 1, Experiment 2 uses the same method to process the data, and finally obtains the combination of eye movement and EEG signals that better judge the cognitive status of operators.

The two experiments performed in this article have a progressive relationship. Experiment 1 considers the operator in normal state, and Experiment 2 is Experiment 1 under special circumstances. The experimental conclusion obtained from Experiment 1 was applied to Experiment 2 and was verified. In Experiment 2, the premise of judging the cognitive status of the operator is to understand the interaction intention of the operator. Therefore, when judging the cognitive status of operators, the horizontal coordinates PX, PY, and saccade amplitude SA were used to judge the interaction intention. At the same time, the EEG signals and eye movement signals were analyzed to analyze the combination of judging the cognitive state.

The experimental results obtained in this article can be applied to the operator human-computer interaction intention prediction and operation state judgment. This article discusses the role of EEG signals and eye movement signals for interaction intention recognition and cognitive state recognition. Compared with the previous research based on eye motor and EEG signals, the human-computer interaction intention is more complex, and the specific characteristics of EEG signals and eye motor signals are matched to the specific human-computer interaction intentions, which are more targeted in the human-computer interaction intention prediction. By measuring the operator’s eye movement and EEG signals, it can match the EEG and eye movement characteristics corresponding to the specific interaction intention, and then realize the prediction of the next operation of the operator. Zhang Qing, Zhao Di, and Tang Lijun’s research on the prediction of human-computer interaction intention realized the discrimination of human-computer interaction intention, but most of their research was based on simple single operation intention, without considering the combination of multiple operation intentions, and almost no researchers considered the prediction of interaction intention of operators in special situations. To sum up, the work done in this article is an in-depth study on the prediction of human-computer interaction intention, and enriches the extended current research orientation, hoping to provide research ideas and help for other researchers. In the next step, we will begin to study the prediction of human-computer interaction intentions of operators in the actual operating environment, consider more complicated operation task interfaces, and apply other physiological signals such as heartbeat, electromyography, and body temperature of operators to the prediction of human-computer interaction intentions, so as to improve the accuracy of the prediction of operation intentions, and enhance the pleasure and operation performance of users in the process of human-computer interaction.

## Data Availability Statement

The raw data supporting the conclusions of this article will be made available by the authors, without undue reservation.

## Ethics Statement

Ethical review and approval was not required for the study on human participants in accordance with the local legislation and institutional requirements. The patients/participants provided their written informed consent to participate in this study.

## Author Contributions

JQ: responsible for method research and data processing. HG: responsible for experimental research. WW: responsible for the correctness verification. SD: responsible for the proofreading of the manuscript. All authors contributed to the article and approved the submitted version.

## Conflict of Interest

The authors declare that the research was conducted in the absence of any commercial or financial relationships that could be construed as a potential conflict of interest.

## Publisher’s Note

All claims expressed in this article are solely those of the authors and do not necessarily represent those of their affiliated organizations, or those of the publisher, the editors and the reviewers. Any product that may be evaluated in this article, or claim that may be made by its manufacturer, is not guaranteed or endorsed by the publisher.
